# Long‐Term High‐Fat Diet Affected Bone Marrow Microenvironment During Aging at Single‐Cell Resolution

**DOI:** 10.1002/mco2.70276

**Published:** 2025-07-21

**Authors:** Yidan Pang, Siyuan Zhu, Peng Ding, Senyao Zhang, Yi Zhang, Fang Ye, Changqing Zhang, Junjie Gao, Jimin Yin

**Affiliations:** ^1^ Department of Orthopaedics Shanghai Sixth People's Hospital Affiliated to Shanghai Jiao Tong University School of Medicine Shanghai China; ^2^ Institute of Microsurgery on Extremities and Department of Orthopedic Surgery Shanghai Sixth People's Hospital Affiliated to Shanghai Jiao Tong University School of Medicine Shanghai China; ^3^ Department of General Surgery Shanghai Sixth People's Hospital Affiliated to Shanghai Jiao Tong University School of Medicine Shanghai China; ^4^ Shanghai Diabetes Institute Shanghai Key Laboratory of Diabetes Mellitus Shanghai Clinical Centre for Diabetes Shanghai Sixth People's Hospital Affiliated to Shanghai Jiao Tong University School of Medicine Shanghai China; ^5^ Center for Stem Cell and Regenerative Medicine Zhejiang University School of Medicine Hangzhou China; ^6^ Liangzhu Laboratory Zhejiang University Hangzhou China

**Keywords:** bone, bone marrow, brain, high‐fat diet (HFD), long‐term HFD to aging (LHA), single‐cell

## Abstract

Obesity and aging are major risk factors for diseases such as Type 2 diabetes mellitus, dementia, and osteoporosis. High‐fat diet (HFD) consumption is one of the most important factors contributing to obesity. To elucidate and provide resources on how long‐term HFD to aging (LHA) affects the bone marrow and solid organs, we established an LHA mice model and demonstrated that LHA caused a shift from osteogenesis to adipogenesis in the bone marrow microenvironment. Single‐cell transcriptomics of bone marrow cells highlighted LHA‐driven perturbations in immune cell populations with distinct metabolic adaptations to LHA. We demonstrated that bone marrow macrophages of the LHA group upregulated Chil3 and Fabp4, which are associated with inflammatory response and regulation of adipocytes. Moreover, we identified the Ptn–Sdc3 axis and Cxcl12–Cxcr4 axis between bone marrow macrophages and brain epithelial cells as possible candidates for crosstalk between bone marrow and brain in LHA mice. Our findings indicated the bone marrow microenvironment as a central hub of LHA‐induced pathology, where adipogenic reprogramming and myeloid cell dysfunction collectively drive skeletal and systematic inflammation. This resource highlights therapeutic opportunities targeting bone marrow to mitigate obesity‐accelerated aging.

## Graphical abstract

High‐fat diet (HFD) promotes obesity and degenerative diseases. We established a long‐term HFD to aging (LHA) mouse model, finding it induces a bone marrow shift from osteogenesis to adipogenesis and abnormal immune cell metabolic adaptations. Signaling axes were identified through single‐cell RNA sequencing as potential bone marrow‐brain crosstalk pathways, providing insights for treating related diseases.

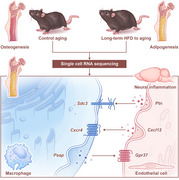

## 1 Introduction

Obesity, marked by an abnormal buildup of body fat, disrupts metabolic balance and significantly increases the likelihood of developing Type 2 diabetes, dementia, osteoporosis, and multiple forms of cancer [1]. Globally, over a third of the population is impacted by overweight or obesity [2]. These conditions, along with hyperlipidemia, are particularly prevalent among middle‐aged and older adults [3]. In China, the prevalence of overweight peaked after age 50 years [4]. As life expectancy rises, the combination of aging and obesity has emerged as a widespread societal concern. A key driver of obesity is the consumption of a high‐fat diet (HFD) [5], making it essential to explore the molecular mechanisms underlying the effects of long‐term HFD on aging (LHA).

HFD leads to an increase in total body fat, including bone marrow fat. The bone marrow serves as a structural and functional foundation, housing specialized microenvironmental niches that play a critical role in maintaining the activity and regulation of immune cells as well as hematopoietic stem cells (HSCs) [6]. Most HSC and immune progenitor cells were found in trabecular bone [7, 8]. Changes in trabecular bone, including compromised osteolineage cells and increased bone marrow fat, can hinder bone regeneration and influence the progeny of bone marrow immune cells in the context of aging and obesity [9, 10, 11]. A recent study indicated that HFD causes bone marrow adipocyte whitening, driving an increase and activation of bone marrow Ly6C^high^ monocytes [12]. Notably, alterations in bone marrow fat composition are specifically associated with long‐term HFD consumption and aging rather than short‐term dietary changes [10].

Moreover, the bone marrow serves as the primary source of immune cell progenitors, including bone marrow‐derived macrophages (BMDMs), which play a critical role in systemic immunity [6]. Notably, changes in the bone marrow can have far‐reaching effects on solid organs such as the brain [13]. Our prior research on aged mice revealed ligand–receptor interactions between myeloid cells in the bone marrow and epithelial cells in the brain, underscoring the significance of bone marrow‐derived cells in the progression of brain degenerative disorders [14]. A more recent study suggested cognitive aging could be reversed by reprogramming myeloid cells derived from bone marrow to restore youthful immune functions [15], highlighting bone marrow–peripheral organ crosstalk. In neurodegenerative conditions like Alzheimer's disease (AD), transplanting young bone marrow into AD mouse models has been shown to mitigate AD‐related pathological changes in the brain [16]. However, less well studied is the impact of LHA on cells in the bone marrow mesenchymal environment, which in turn directly influences and regulates immune cell functions [6]. An examination of the molecular changes induced by LHA during aging across bone, bone marrow, and solid organs is critical to understanding how LHA reshapes bone, influences bone marrow function, and contributes to systematic injury during aging.

In our investigation, we observed that LHA was associated with bone loss and elevated bone marrow adiposity. Utilizing single‐cell RNA sequencing (scRNA‐Seq), we further explored the cellular diversity and functional states of bone marrow immune cells, revealing widespread disruptions in lipid metabolism and inflammation within bone marrow cell populations. Additionally, scRNA‐Seq of the LHA brain uncovered evidence of neural injury, potentially linked to interactions between bone marrow immune cells and brain epithelial cells.

## 2 Results

### 2.1 Phenotypic Response to LHA in Mouse Model

**FIGURE 1:**
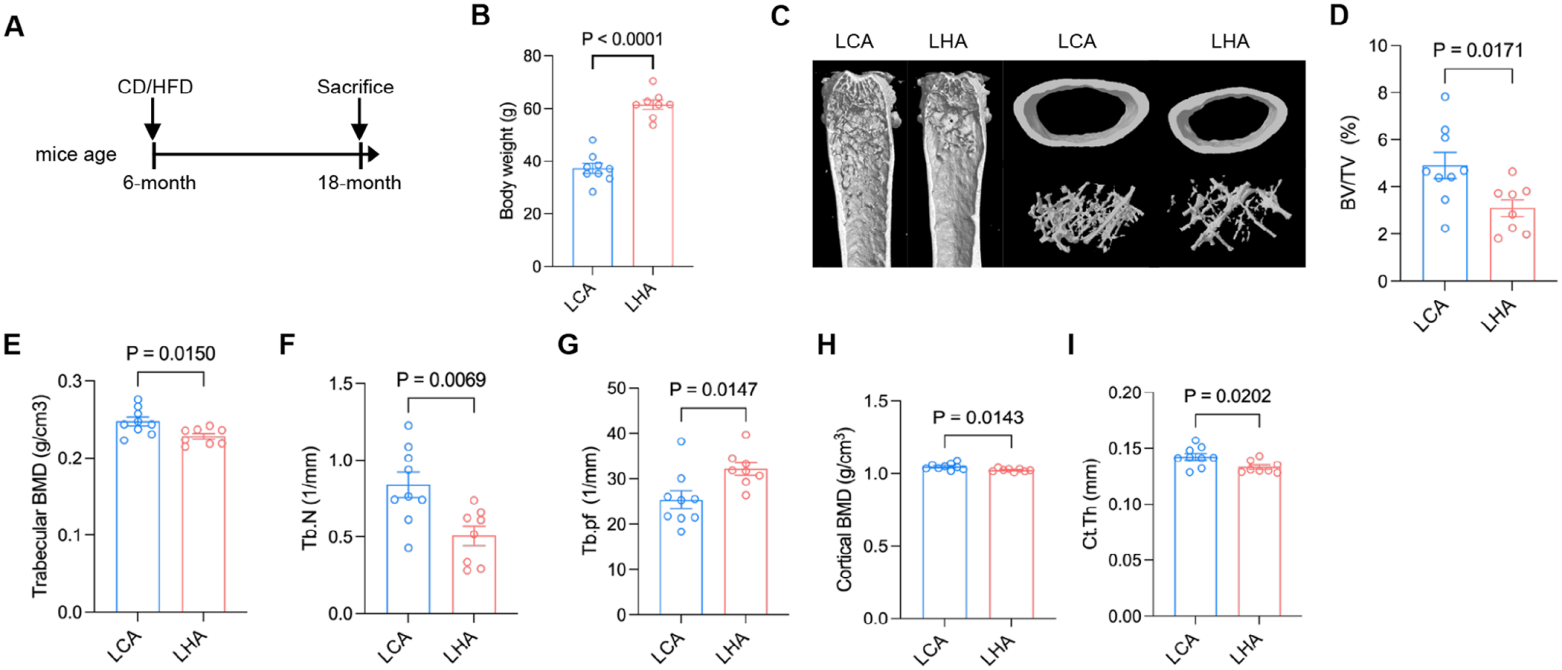
Phenotypic response of LHA mice to aging. (A) Schematic diagram depicting experimental setup of animal study. (B) Body weight of mice from the LCA and LHA groups at the time of sacrifice. (C) Representative micro‐computed tomography (µCT) reconstructive images of LCA and LHA mice femur aged 18 months, and (D–G) trabecular microstructural parameters (BMD, bone mineral density; BV/TV, bone volume fraction; Tb.N, trabecular number; Tb.Pf, trabecular bone pattern factor) and (H, I) cortical microstructural parameters (BMD; Ct.Th, cortical thickness) derived from µCT analysis. Data are represented as mean ± SEM. Each dot represents a biological replicate (*n* = 9 for the LCA group and *n* = 8 for the LHA group).

To validate the phenotypical association between bone and brain during aging, we established 18‐month‐old C57BL/6J male mice of LHA and LCA feeding (Figure 1A). LHA mice induced a weight gain of a 1.5‐fold increase compared to LCA (Figure 1B).

Moreover, micro‐CT analysis demonstrated LHA‐induced bone loss in aged mice (Figure 1C), as indicated by a decrease in bone mineral density (BMD), bone volume fraction (bone volume/tissue volume %, [BV/TV %]), trabecular number (Tb.N), and trabecular bone pattern factor (Tb.Pf) of femur trabecular bone (Figure 1D–G). Cortical BMD and cortical thickness (Ct.Th) showed a significant decrease (Figure 1H–I). Those results indicate a compromised bone microenvironment for bone marrow accompanied by weight gain due to LHA.

### 2.2 Tissue‐Level Transcriptome Perturbation of Bone Marrow Mesenchymal Environment in LHA

**FIGURE 2:**
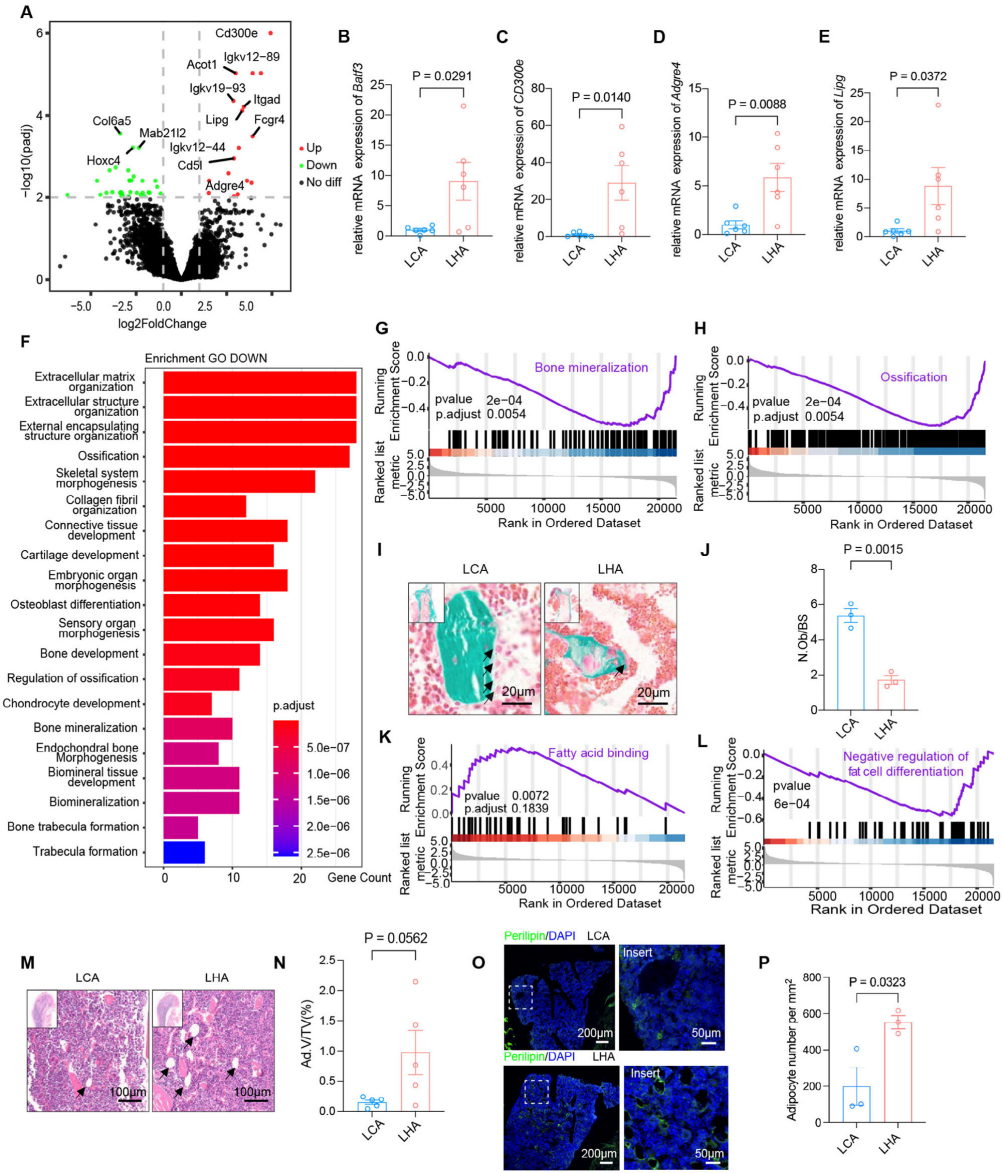
Tissue‐level transcriptome perturbation of LHA in bone marrow mesenchymal environment. (A) Volcano plot of differentially expressed genes (DEGs) from bulk RNA‐seq of bone particle (767 upregulated, 803 downregulated, |log2FoldChange| > 2). (B–E) RT‐qPCR verification of DEGs. *n* = 6 for each group. (F) GO pathway analysis of downregulated genes. (G, H) Gene Set Enrichment Analysis (GSEA) of bone mineralization and ossification. (I) Goldner's trichrome stain of mice trabecular bone and (J) histomorphometry analysis of osteoblast numbers per bone surface (N.Ob/BS) (arrows). *n* = 3 for each group. (K, L) GSEA of fatty acid binding and negative regulation of fat cell differentiation. (M) Hematoxylin–eosin (H&E) stain of mice femur bone marrow and (N) histomorphometry analysis of adipocyte (arrows) volume per tissue volume (Ad.V/TV). *n* = 5 for each group. (O) Immunofluorescence staining of perilipin^+^ cells in bone marrow and (P) quantification of perilipin^+^ cell numbers per area. *n* = 3 for each group. Data are represented as mean ± SEM. Each dot represents a biological replicate.

To examine the molecular base underlying the LHA‐induced bone phenotype, we conducted bulk RNA sequencing (bulk RNA‐seq) of the bone particles to explore the regulation of the bone marrow mesenchymal environment in LHA mice and identified 1570 differentially expressed genes (DEGs) (767 upregulated, 803 downregulated, |log2FoldChange| > 2). We observed upregulation of myeloid cell function differentiation‐related genes (*Cd300e*, *Adgre4*) [17, 18] and dendritic cell transcription factor *Batf3* (Figure 2A) [19], which were determined by reverse transcription‐polymerase chain reaction (RT‐PCR) (Figure 2B–D).

Gene ontology (GO) pathway enrichment revealed activation of immune response and myeloid cell antigen processing via MHC‐I (Figure S1A). Myeloid cells give rise to osteoclasts, which develop and adhere to bone matrix, then secrete acid and lytic enzymes that degrade it [20]. However, osteoclast‐related genes show no significant increase in the LHA group (Figure S1B).

We then assessed the effects of LHA on osteoclastogenesis during aging. BMDMs from LHA and LCA mice of 18 months were collected and plated at the same density for the examination of osteoclastogenesis in vitro. BMDMs from the LHA group also showed no significant difference in osteoclast differentiation capacity as indicated by osteoclast number (Figure S1C,D) and expression of the signature genes of osteoclasts (Figure S1E–J). Similarly, tartrate‐resistant acid phosphatase (TRAP) staining shows no difference in osteoclast numbers (N.Oc/BS) and surface (Oc.S/BS) between LHA and LCA groups (Figure S1K–L).

On the other hand, bone is a dynamic tissue undergoing constant remodeling that is orchestrated by osteoblast‐mediated bone formation and osteoclast‐mediated bone resorption [21]. GO pathway analysis of downregulated genes demonstrated enrichment in ossification, osteoblast differentiation, and trabecular bone formation (Figure 2F), and Gene Set Enrichment Analysis (GSEA) also revealed downregulation of bone mineralization (Figure 2G) and ossification (Figure 2H). We further analyzed the expression of the ossification‐related gene module. Gene expression heatmap also demonstrated significant downregulation of ossification genes (Figure S1N). Goldner's trichrome stain indicated a decrease in osteoblast number (Figure 2I,J). These results indicated a reduced bone formation activity by osteoblasts under LHA. Bone is a dynamic tissue undergoing constant remodeling that is orchestrated by osteoblast‐mediated bone formation and osteoclast‐mediated bone resorption [21], but our previous results showed no significant changes in osteoclast activity. Thereby, LHA‐related bone loss was mainly caused by a reduction in osteoblast‐mediated bone formation.

Moreover, GSEA also revealed upregulation of fatty acid binding (Figure 2K) and fat cell differentiation (Figure 2L). Upregulation of *Lipg*, a gene related to fatty acid binding, was also determined by RT‐qPCR (Figure 2E). Intriguingly, hematoxylin–eosin (H&E) stain showed increasing adipocyte volume (Ad.V/TV) in the femur bone marrow trabecular area (Figure 2M,N), and immunofluorescence staining of perilipin showed an increase in adipocyte number (Figure 2O,P). Also, further analysis of fat cell differentiation‐related gene modules showed enrichment of fat cell differentiation genes (Figure S1O). These results indicating a shift from osteogenesis to adipogenesis contributed to bone loss in LHA rather than osteoclastogenesis.

### 2.3 Cellular Heterogeneity of Bone Marrow Cells After LHA

**FIGURE 3:**
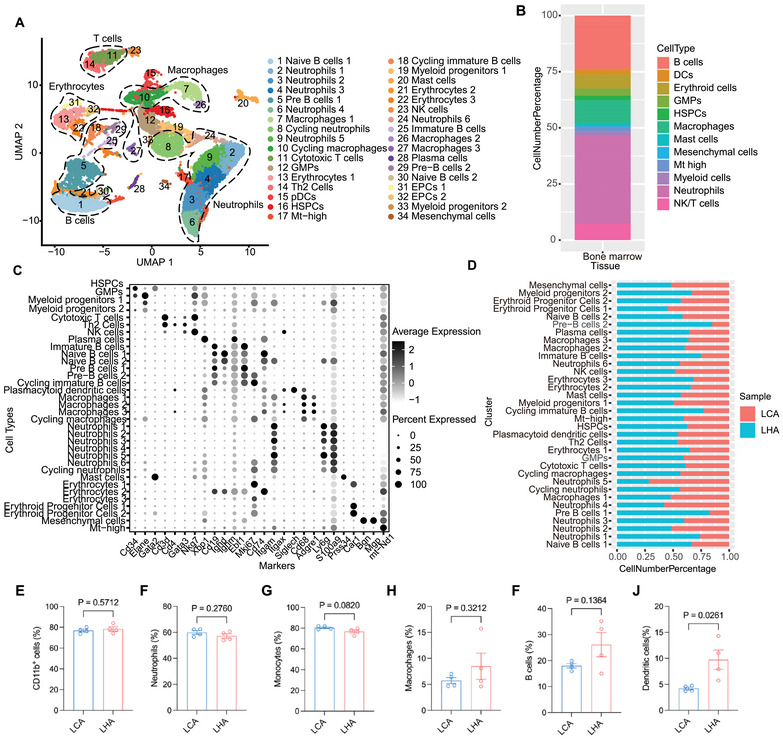
Cellular heterogeneity and single‐cell level gene alterations of bone marrow by LHA. (A) UMAP plots showing the cell clusters in merged bone marrow samples. (B) Bar plots showing the ratio of major cell types in merged bone marrow samples. (C) Dot plots showing the expression of marker genes in merged bone marrow samples. (D) Bar plots showing the ratio of each cell cluster in different conditions in merged bone marrow samples. (E–J) Flow cytometry analysis of proportions of CD11b^+^ cells, neutrophils, monocytes, macrophages, B cells, and dendritic cells in the bone marrow of CD and HFD mice. *n* = 4 for each group. Data are represented as mean ± SEM. Each dot represents a biological replicate.

To decipher the influence of LHA on cellular composition and functional states of bone marrow, we further characterized single‐cell transcriptome profiling of bone marrow in two groups. After filtering low‐quality cells with few transcripts and a high number of mitochondria genes (Figure S2A), we identified 19 major cell types in UMAP (Uniform Manifold Approximation and Projection) clusters of bone marrow samples (Figure 3A). A high proportion of neutrophils and other myeloid cells was observed (Figure 3B). Cell types in bone marrow samples included *Cd34* ^+^ hematopoietic stem and progenitor cells (HSPCs); granulocyte‐monocyte progenitors (GMPs, *Cd34* ^+^ and *Elane* ^+^); neutrophils (*Ly6g* ^+^); macrophages (*Cd68* ^+^, *Adgre1* ^+^); B cells (*Cd19^+^*); and other cell types, including Th2 cells (*Cd3* ^+^, *Cd4* ^+^, *Gata3^+^*), cytotoxic T cells, NK cells, plasmacytoid dendritic cells (*Siglech* ^+^), and mast cells (*Prss34* ^+^) (Figure 3C). HFD condition‐derived bone marrow samples occupied a large amount of pre‐B cells and immature B cells (Figure 3D). To corroborate the results of scRNA‐seq, flow cytometry of major cell types in bone marrow was performed (Figure 3E–J and Figure S2C). Flow cytometry analysis revealed that there was a slight increase in B cells and dendritic cells of the HFD group at 18 months compared to the LCA group (Figure 3I,J).

### 2.4 Bone Marrow Single‐Cell Level Gene Alterations by LHA

**FIGURE 4:**
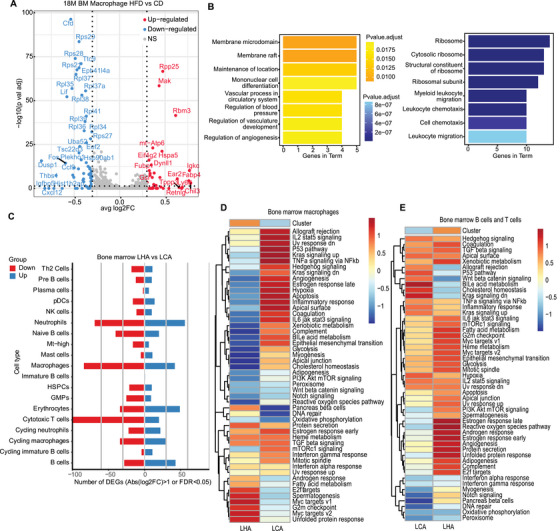
DEGs pathway analysis in LHA bone marrow. (A) Volcano plots (left) and (B) gene function enrichment results of DEGs (right, upregulated: yellow; downregulated: blue) in bone marrow macrophages. (C) Bar plots showing the number of DEGs in bone marrow in LHA condition. (D) GSVA enrichment results of bone marrow macrophages (E) bone marrow lymphocytes.

We analyzed DEGs in different cell types from bone marrow and brain samples in LCA and LHA. Cell types with certain levels of DEGs were selected for GO enrichment analysis. In bone marrow macrophages, the LHA group upregulated *Chil3* and *Fabp4* (Figure 4A). The enriched terms included mononuclear differentiation and angiogenesis (Figure 4B). A higher level of *Fabp4* in LHA mice was associated with inflammatory response and adipocyte regulation [22, 23]. We confirmed the upregulation of *Fabp4* in BMDMs from the LHA group (Figure S3A). Accordingly, we detected serum levels of inflammatory cytokines by bead‐based immunoassay (Figure S3B–K). The LHA group serum showed a significantly higher level of interleukin‐6 (IL‐6) and interleukin‐12p70 (IL‐12p70) (Figure S3A,B). IL‐6 is produced in response to infections and tissue injuries and plays different roles in different contexts, while dysregulated continual synthesis of IL‐6 has a pathological effect on chronic inflammation and autoimmunity [24]. IL‐12p70 is mainly produced by activated monocytes, macrophages, and DCs [25]. Interleukin‐12 (IL‐12) is essential for the differentiation and maintenance of Th1 effector cells, which link the innate immune system to the adaptive immune system [26].

In the bone marrow, T cells, neutrophils, and macrophages harbored the greatest number of DEGs across all the cell types in the LHA condition (Figure 4C). We performed gene set variation analysis (GSVA) of specific pathways in different conditions. In LHA bone marrow macrophages, unfolded protein response (UPR)‐induced UPR and MYC target pathways were enriched (Figure 4D). LHA‐induced ER stress triggered the UPR pathway and may decrease protein synthesis as well as macrophage polarization [27, 28] (Figure 4D). In lymphocytes (B cells and T cells), LHA lymphocytes enriched reactive oxygen species (ROS), which could lead to bone marrow suppression [29] (Figure 4E). Interestingly, neither macrophages nor lymphocytes of bone marrow demonstrated a higher enrichment level of acute inflammatory response (Figure 4D,E), as reported in short‐term HFD [30]. Meanwhile, the overall level of serum also shows no upregulation of inflammatory cytokines such as interleukin‐1 (IL‐1) and tumor necrosis factor‐α (TNF‐α) (Figure S4C–J). It could possibly be attributed to the dysfunction of the bone marrow immune system after LHA.

### 2.5 Overlap of LHA Mice and Osteocyte‐Deficient Mice Gene Perturbation in Bone Marrow

Specialized niches within the bone marrow guide and constrain the development of HSCs and lineage‐restricted immune progenitor cells. Trabecular bone, especially the osteolineage cells, contributes to the niche. We have previously reported that conditional deletion of osteocytes by the expression of diphtheria toxin subunit α in osteocytes caused a shift from osteogenesis to adipogenesis of the mesenchymal environment and mobilized myeloid lineage differentiation in bone marrow [9]. The imbalanced adipo‐osteogenic differentiation caused by the partial ablation of osteocytes paralleled the LHA condition (Figure 2). We also observed a reduction in the osteocyte number of LHA femurs (Figure S4A,B). Therefore, we further evaluated the overlap perturbation genes between osteocyte‐deficient mice and LHA bone marrow samples to narrow down the perturbation caused by the mesenchymal environment. LHA bone marrow samples demonstrated several overlapping upregulated and downregulated genes (Figure S4C,D). We checked the expression patterns of those overlapping genes in myeloid cells. *Ms4a6c*, *Ctss*, *Ccr2*, and *Ccl9* were enriched in macrophages (Figure S4E). Functional enrichment of overlapped upregulated genes indicated antigen processing and presentation and positive regulation of immune response (Figure S4G). Downregulated genes, including *Pla2g7*, *Oast2*, and *Pbx1*, were enriched in neutrophils with the function of lymphocyte activation and cytokine production (Figure S4F,H). These results suggested that LHA may induce similar perturbations in bone marrow macrophages compared with the osteocyte ablation condition, which is supportive of the regulation of the mesenchymal environment on bone marrow macrophages during LHA.

### 2.6 Single‐Cell Level Gene Alterations of Brain by LHA

**FIGURE 5:**
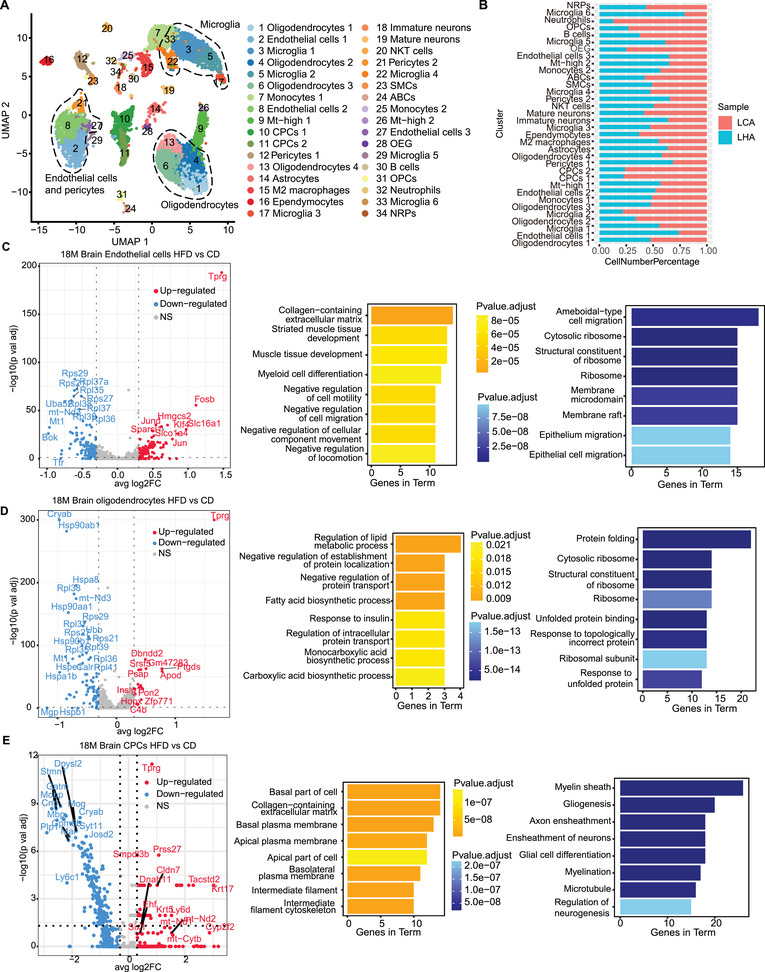
Single‐cell level gene alterations of the brain by LHA. (A) UMAP plots showing the cell clusters in merged brain samples. (B) Bar plots showing the ratio of each cell cluster in different conditions in merged brain samples. (C–E) Volcano plots (left) and gene function enrichment results of DEGs (right, upregulated: yellow; downregulated: blue) in brain endothelial cells (C), brain oligodendrocytes (D), and CPCs (E).

To explore the potential regulatory relationship between bone marrow and solid organs, we extended our analysis to the brain to investigate potential interactions by conducting scRNA‐Seq of the brain on the same batch of LHA and LCA mice. After filtering low‐quality cells with few transcripts and a high number of mitochondria genes in brain samples (Figure S2B), we annotated 18 major cell types (Figure 5A,B). Different glial cells dominated the majority of cells detected (Figure 3D). We identified *Pdgfra* ^+^ oligodendrocyte precursor cells (OPCs); oligodendrocytes (*Cldn11* ^+^ and *Mog* ^+^); microglia (*Tmem119* ^+^); endothelial cells (*Cldn5* ^+^); pericytes (*Kcnj8* ^+^); choroid plexus epithelial cells (CPCs, *Ttr* ^+^); astrocytes (*Gja1* ^+^); ependymocytes (*Ccdc153* ^+^); arachnoid barrier cells (ABCs, *Slc47a1* ^+^); immature neurons (*Sox11* ^+^); mature neurons (*Syt1* ^+^); and immune‐related cell types (Figure S5A). HFD‐derived brain samples showed a reduced proportion of CPCs, neutrophils, and OPCs (Figure 5B).

In the brain, oligodendrocytes, microglia, CPCs, and endothelial cells demonstrated a high number of upregulated DEGs in the LHA condition (Figure S5B). Results from GO enrichment showed inhibition of cell migration in the LHA group endothelial cells, indicating a compromised capacity to restore vessel integrity (Figure 5C). LHA group oligodendrocytes showed an increased abundance of *Apod* in the elderly and Alzheimer's dementia (Figure 5D) [31]. Enriched GO terms in oligodendrocytes included fatty acid biosynthetic process and lipid metabolic process (Figure 5D). In CPCs, we identified remarkable downregulation of *Plp1*, *Mobp*, *Mbp*, and *Stmn1*, which were associated with gliogenesis and neurogenesis in the LHA condition (Figure 5E) [32]. Generally, myeloid cell differentiation was induced in LHA bone marrow samples. LHA condition induced reduction of vascular cell subtypes in the brains of aged mice as well as their regulation function of gliogenesis and neurogenesis. Further GSVA analysis showed LHA‐induced interferon alpha response in oligodendrocytes (Figure S5C) as well as oxidative phosphorylation and DNA repair in microglia. In LHA microglia, hypoxia and IL2‐STAT5 signaling pathways were enriched (Figure S5D). LHA‐induced inflammation and oxygen‐deficient environment in brain glial cells may further contribute to neuroinflammation [33].

We next validated if neurological injury and neural inflammation coexist with the mentioned bone marrow changes in our model. Neurons of the hippocampus in the LCA group displayed integrative and well‐maintained morphology by Nissl staining (Figure S5E). However, in the LHA group, the morphology of neurons in the dentate gyrus (DG), CA1, and CA3 appeared indistinct, and their Nissl substance decreased compared with the LCA group (Figure S5E). The number of viable neurons was significantly reduced in the LHA group (Figure S5F–G), which indicated a low functional state of hippocampal neurons in LHA mice. In previous studies, microglia activation was reported to mediate HFD‐induced neuroinflammation [34, 35], and microglia dysfunction contributed to the neurological injury [36, 37]. In LHA mice, a significant increase in Iba1^+^ microglia number was detected in the hippocampus (Figure S5I,J), which indicated an activation of hippocampal microglia. However, the percentage of CD68^+^ cell numbers in Iba1^+^ microglia decreased in the LHA group (Figure S5K), indicating decreased phagocytic activity of microglia. Although the number of microglia increased due to the stimulation of HFD, the function of microglia might be compromised by LHA. These results indicated the underlying connection between impaired bone marrow microenvironment and brain pathology.

### 2.7 Intercellular Communication Between Bone Marrow Macrophages and Brain Vascular Cells

**FIGURE 6:**
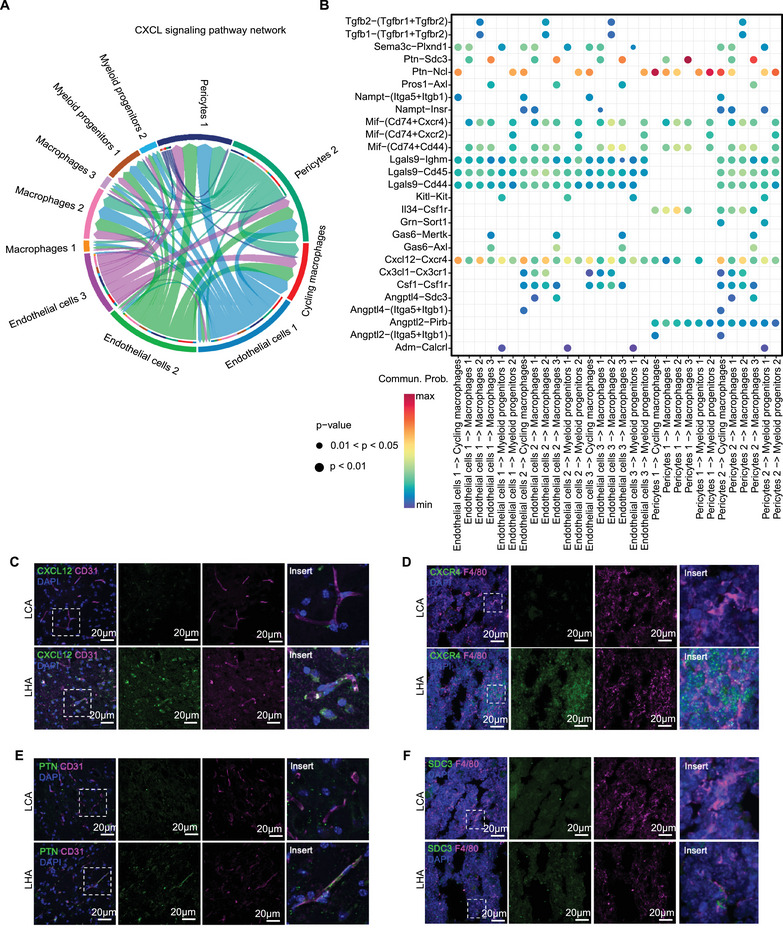
Gene regulation network between LHA bone marrow and brain. (A) Cell–cell interaction network of CXCL signaling pathways between bone marrow myeloid cells and brain stromal cells (directions indicated the ligand–receptors from brain to bone marrow). (B) Heatmap showing the specific ligand–receptor pairs in (A). (C) Immunofluorescence staining of CXCL12 in brain CD31^+^ endothelial cells. (D) Immunofluorescence staining of CXCR4 in bone marrow F4/80^+^ macrophages. (E) Immunofluorescence staining of PTN in brain CD31^+^ endothelial cells. (F) Immunofluorescence staining of sydecan‐3 (SDC3) in bone marrow F4/80^+^ macrophages.

To evaluate the LHA‐induced inflammation crosstalk between bone marrow macrophages and brain vascular cells. We constructed a cell–cell communication network of brain vascular cells (endothelial cells and pericytes) and bone marrow macrophages in LHA conditions using CellChat [38] (Figure 6A). The *Ptn–Sdc3* axis played a pro‐migratory and pro‐differentiating role on cortical neurons [39] (Figure 6B). Endothelial cells expressing *Ptn* could interact with *Ncl* to promote cell migration [40, 41]. The *Cxcl12–Cxcr4* axis also contributed to the migration of bone marrow‐derived peripheral macrophages through the blood‐brain barrier (BBB) at LHA condition (Figure 6B). It could be inferred that bone marrow macrophages were recruited via the *Ptn–Sdc3* axis and *Cxcl12–Cxcr4* axis. We further validated our RNA sequencing results by immunofluorescence staining. We observed increased expression of CXCL12 in brain CD31‐positive endothelial cells, alongside an upregulation of CXCR4 in bone marrow macrophages (Figure 6C,D). An increase in PTN expression in brain CD31‐positive endothelial cells and Sdc3 expression in bone marrow macrophages was also observed (Figure 6E,F).

To explore the ligand–receptor interactions from bone marrow macrophages to brain vascular cells, we constructed a cell–cell communication network starting from bone marrow myeloid cells to brain vascular cells (endothelial cells and pericytes) in LHA condition using CellChat (Figure S6A). The *Psap‐Gpr37* axis was identified as a candidate with the highest probability in the LHA group, which mainly links bone marrow macrophages to endothelial cells subpopulation 3 (Figure S6B). Prosaposin (PSAP), a highly conserved glycoprotein, is a neurotrophic factor, as well as a regulator of lysosomal enzymes [42]. The orphan G‐protein‐coupled receptors GPR37 were recognized as PSAP receptors to mediate their neuroprotective roles in neurons [43]. Intriguingly, in atherosclerosis models, which is also a lipid‐induced chronic inflammatory condition, downregulation of PSAP secretion in macrophages contributes to vascular inflammation [44]. PSAP presents as an anti‐inflammatory factor secreted by plaque macrophages in the apolipoprotein E deficient (Apoe^−/−^) mice model [44]. Upon our analysis, it was supportive that the *Psap‐Gpr37* axis could mediate the protective role of bone marrow macrophages on brain stromal cells.

### 2.8 Gene Regulation Network of LHA Bone Marrow and Brain

We further constructed transcriptional factor regulation programs of potential interaction cell types in LHA bone marrow and brain using SCENIC [45]. In LHA bone marrow, B cell maturation was promoted by the enrichment of *Erg* (Figure S6C). Cycling immature B cells and pre‐B cells enriched cell cycle regulators, including *Brca1*, *Rad21*, *E2f7*, and *E2f8*. Macrophages exhibited high activity of differentiation factor Mafb. Besides, the regulon activity of *Nr1h3* (*Lxrα)* in macrophages played a role as fatty acid sensors and regulated cholesterol homeostasis [46]. The activated *Nr1h3* pathway in macrophages induces expression of transporter *Apoe* to initiate the process of reverse cholesterol transport in LHA‐induced inflammation environments [46]. The other transcriptional mediators of inflammatory signals, *Nr4a1* and *Pparg*, may also contribute to the inflammation process in bone marrow macrophages.

In the brain, endothelial cells showed enrichment of the regulon Fosl1, which is related to angiogenesis in the LHA hypoxia state (Figure S6D) [47]. The activation of Nfe2l2 (*Nrf2*) in microglia shields cells from oxidative stress caused by hypoxia and reduces neuroinflammation [48]. In the brain, oligodendrocytes exhibit an abundance of myelination‐promoting factors (such as *Sox10*) alongside inhibitory regulators (like *Sox2*), which could contribute to prolonged neurodevelopmental impairments through axonal degeneration [49]. These results of transcriptional factor regulation events support altered lipid metabolism in LHA bone marrow macrophages and hypoxia, oxidative stress, and neuroinflammation in brain glial cells.

## 3 Discussion

HFD is commonly used to model obese mice. HFD is related to decreased bone mass and an increased risk of cognitive impairment [50, 51] and also stimulates innate immune cells, leading to a transient inflammatory response [52]. However, the interplay between bone marrow immune cells and the brain remains poorly understood in the context of LHA. In this study, we examined how the bone marrow mesenchymal environment and immune system in LHA mice contribute to alterations in cell type populations, gene expression patterns, and cell–cell communications with bone marrow immune cells. Overall, LHA exacerbates hypoxia‐induced oxidative stress during aging, leading to degenerative changes and neural inflammation in the brain. Increased bone marrow adiposity and impaired ossification may contribute to dysfunction in bone marrow immune cells, potentially exacerbating neuroinflammation.

Duration and timing of HFD exposure critically shape metabolic outcomes. Short‐term HFD exposure (days to weeks) primarily induces acute metabolic disturbances. Studies highlight transient insulin resistance, particularly in the liver and skeletal muscle, driven by lipid accumulation and mitochondrial stress [53]. However, prolonged HFD will lead to functional and structural changes due to maladaptation, such as adipose tissue remodeling, alongside β‐cell dysfunction, atherosclerosis, osteoporosis, and neuronal inflammation, contributing to irreversible metabolic syndrome [54]. In current rodent models, HFD shows a different influence on both skeletal metabolism and cognitive function, which is associated with HFD duration and animal age. HFD with > 50% of energy from fats and an intervention time of 10 weeks to 5 months are more likely to induce skeletal alterations [50]. HFD that lasts longer than 4 months can induce neuroinflammation and cognitive impairment in young and adult rodents [55, 56, 57], while aged rodents have hippocampal and amygdala‐based cognitive deficits after only 3 days of HFD [51]. However, the effect of LHA has not been studied in rodent models.

We identified several upregulated genes related to lipid metabolism in different cell types across tissue, which indicated an overall perturbation of lipid metabolism, such as upregulated *Fabp4* in bone marrow macrophages (Figure 4A), *Apod* in oligodendrocytes (Figure 4E), and *Lipg* in cortical bone (Figure 2A). Fatty acid binding protein 4 (FABP4) is an intracellular lipid chaperone and adipokine that plays a key role in fatty acid transport and macrophage‐mediated inflammation, which activates pathways such as peroxisome proliferator‐activated receptor gamma and IkappaB kinase, contributing to inflammatory responses [58]. Studies in HFD mouse models have demonstrated that deleting FABP4 in macrophages reduces inflammation and provides protection against obesity‐related metabolic dysfunction [59]. Apolipoprotein D (APOD), a member of the lipocalin family, functions as a transporter for hydrophobic molecules, including sterols, steroid hormones, and arachidonic acid. It is expressed in both peripheral and neural tissues, with significant levels found in OPCs [60]. Research suggests that APOD expression increases in stressed cortical neurons during aging and in AD, potentially preceding the formation of neurofibrillary tangles [31]. Elevated APOD levels in oligodendrocytes may also play a role in the cognitive impairments associated with HFD. Endothelial lipase (LIPG), a cell surface‐associated enzyme with phospholipase A1 activity, primarily targets phosphatidylcholine in high‐density lipoproteins (HDL) [61]. Increased LIPG expression in cortical bone suggests a systemic influence of lipid metabolism on the bone marrow microenvironment (Figure 2A). During HFD, LIPG may promote lipid accumulation in bone marrow stromal cells, while elevated circulating free fatty acids could impair lipid processing mechanisms across various tissues.

LHA interfered with the redox state in brain glial cells and epithelial cells (Figure 5). In our study, the *Ptn–Sdc3* axis and *Cxcl12–Cxcr4* axis were distinguished as activated axes between brain epithelial and bone marrow macrophages (Figure 6A,B). In cerebral ischemia‐reperfusion injury, the pleiotrophin (PTN)/syndecan‐3 pathway mediates the neuroprotective effect in heparin therapy [62]. The *Cxcl12–Cxcr4* axis plays a critical role in the recruitment of monocytes and the promotion of M2 macrophage polarization [63]. In the context of cognitive impairment linked to neuropathic pain, CXCL12 facilitates the migration of monocytes into the brain's perivascular spaces, contributing to memory deficits [64]. Similarly, in triple‐negative breast cancer, this axis drives the recruitment of monocytes to tumor sites, where they differentiate into a specific subpopulation of lipid‐associated macrophages characterized by STAB1 and TREM2 expression, which exhibit immunosuppressive properties [65]. Additionally, the *Cxcl12–Cxcr4* axis is implicated in the activation and migration of BMDMs into the brain, highlighting its broad influence on immune cell dynamics across various physiological and pathological conditions. Additionally, we identified the *Psap‐Gpr37* axis as a communication pathway through which bone marrow macrophages interact with the brain (Figure S5B). PSAP, a neuroprotective agent, interacts with the GPR37 receptor [43]. Studies in mice indicated that mutations in *Psap* correlate with the onset of neurodegenerative conditions [66]. Additionally, PSAP exhibits anti‐inflammatory properties in mediating interactions between arterial endothelial cells and plaque macrophages [44], implying it may play a comparable role in the relationship between bone marrow macrophages and endothelial cells. Our findings on the interaction between bone marrow and brain suggested that bone marrow macrophages play a dual role, acting as both mediators of neural inflammation and potential regulators of vascular inflammation within specific endothelial cell subpopulations, such as endothelial cells 3 (Figure S5B), in LHA mice [67]. Thereby, deciphering specific ligand and receptor interactions between bone marrow macrophages and endothelial cells helps the identification of possible neuroprotective factors.

The early onset of chronic diseases in obesity parallels those of aging, such as cognitive deficit and osteoporosis [68, 69]. We have observed a shorter lifespan during aging in LHA mice (Figure 1D). This reduced life expectancy may be attributed to obesity accelerating aging processes through immune system impairment. Given that immune cells can be influenced by the bone marrow mesenchymal environment, the altered immune system observed in LHA mice could potentially be linked to changes in the mesenchymal environment [9, 10, 11]. We identified several commonly perturbed genes in bone marrow macrophages of LHA mice and under osteocyte‐deficient conditions (Figure S2C–F). Moreover, it is well‐established that conditions characterized by intense and prolonged stimulation often result in adverse outcomes, such as low‐grade chronic inflammation and dysfunction or decline of immune cells [37]. Aging is characterized by chronic inflammation and dysfunction of the immune system [70]. We revealed that bone marrow immune cells in LHA mice are different from those in HFD, as reported in previous studies [30]. Age‐related immunodeficiency exacerbated by an HFD can lead to dysregulation in peripheral immunity, as indicated by enriched UPR and ROS pathways in LHA bone marrow macrophages and lymphocytes (Figure 5D). This dysregulation may impair the ability of peripheral immune cells to appropriately respond to BBB disruption and neuroinflammation in LHA. For instance, the protective roles of bone marrow macrophages on brain endothelial cells via the *Psap‐Gpr37* axis could be compromised due to macrophage dysfunction (Figure S5B).

While this work provides novel insights into the bone marrow microenvironment under long‐term HFD conditions, several limitations warrant consideration. First, the study utilized aged male mice to model aging and obesity, which may limit generalizability to female populations or younger cohorts, as sex‐ and age‐specific hormonal variations could influence metabolic and skeletal responses to HFD. Second, although scRNA‐Seq and validation were employed to illustrate key pathways like *Cxcl12–Cxcr4*, causal mechanistic links between bone marrow dysfunction and solid organ disease remain partially inferred; future studies using conditional knockout models or in vivo pathway inhibition would strengthen these conclusions. Finally, the HFD composition (e.g., specific lipid profiles) and its duration, while standardized, may not fully recapitulate human dietary patterns or chronic disease progression. Addressing these limitations in future work will refine our understanding of HFD‐driven bone marrow remodeling and its therapeutic implications.

## 4 Conclusion

Our study presents a detailed transcriptome profile of the bone marrow mesenchymal environment, bone marrow immune cells, and whole brain in aged LHA mice. We identified significant transcriptional changes in bone cells, bone marrow macrophages, and brain CPCs, along with their possible interactions. These findings serve as a valuable resource for understanding the impacts of LHA during aging and may aid in developing new approaches for treatment and prevention of significant societal burdens.

## 5 Materials and Methods

### 5.1 Animals

Adult male C57BL/6J mice at 6 months of age (Beijing Vital River Laboratory Animal Technology Co. Ltd.) were fed a 60% fat diet (D12492, Research Diets) or standard chow (11% fat) for 12 months. The mice were housed under specific pathogen‐free conditions at 22°C–24°C and on a 12‐h/12‐h light/dark cycle. Isoflurane was administered prior to the sacrifice of the animals.

### 5.2 Bone Density Measurements

The mouse femur was dissected and fixed in 4% paraformaldehyde (PFA) for 24 h and then stored in 70% ethanol until scanned using the µCT instrument (SkyScan 1176). Relevant structure parameters of the µCT instrument were set up as follows: source voltage, 50 kV; source current, 450 µA; AI 0.5 mm filter; pixel size, 9 µm; rotation step, 0.4°C. CTAn micro‐CT software version 1.13 (Bruker) was used to analyze the images. The volumetric regions for trabecular analyses include the secondary spongiosa located 1 mm from the growth plate and extending 1.8 mm (200 sections) proximally. For cortical bone analysis, the volumetric regions include 600 µm long at mid‐diaphysis of the femur (300 µm extending proximally and distally from the diaphyseal midpoint between the proximal and distal growth plates). Morphometric parameters, including BMD, BV/TV, Tb.N, Tb.Pf, and Ct.Ar/Tt.Ar, were calculated.

### 5.3 Histological Analysis

The femurs of mice were dissected and fixed in 4% PFA for 24 h, then decalcified with 10% EDTA at room temperature for 5 days. After decalcification, the samples were embedded in paraffin and sectioned to a thickness of 5 µm. TRAP staining was used to analyze osteoclasts, while H&E staining was used to examine adipocytes and osteocytes. For osteoblast analysis, the undecalcified tibia was embedded in plastic, sectioned at 5 µm thickness, and stained with Goldner trichrome. Histomorphometric analysis was conducted using BioQuant Osteo software (BioQuant), and the number of osteocyte lacunae was measured with ImageJ.

Mice brains were dissected and fixed in 4% PFA for 24 h. The sections were washed twice for 15 min in 0.01 M PBS and incubated in 0.1% toluidine blue staining solution for 30 min at 60°C. Following incubation, the sections were washed with distilled water and dehydrated gradually in ethanol (70%, 95%, and 100%). After dehydration, it was placed in xylene and cover‐slipped using neutral resins. The cells in the DG, CA1, and CA3 regions of the hippocampus were analyzed using the ImageJ analysis program.

### 5.4 BMDM Culture and In Vitro Osteoclastogenesis Assay

Bone marrow cells were harvested by flushing the femurs and tibias of mice. These cells were cultured at 37°C in a humidified incubator with 5% CO_2_ using α‐MEM (MeilunBio) supplemented with 10% FBS (Gibco), 100 µg/mL streptomycin (Gibco), 100 U/mL penicillin (Gibco), and 30 ng/mL recombinant murine M‐CSF (PeproTech). The medium was changed every 2 days. After 5 days, BMDMs were either collected for RNA extraction or seeded into 96‐well and 12‐well plates. An additional 5 days of culture with 30 ng/mL M‐CSF (PeproTech) and 100 ng/mL RANKL (PeproTech) was done before tartrate‐resistant acid phosphatase (TRAP) staining. Cells were then fixed and stained for TRAP as per the manufacturer's instructions (Sigma) to quantify osteoclasts. Mature osteoclasts were identified as TRAP‐positive cells with more than three nuclei. RNA extraction was performed accordin g to the recommended protocol when needed.

### 5.5 RT‐qPCR

The EZ‐press RNA Purification Kit PLUS was employed for total RNA extraction. Subsequently, 4 × EZscript Reverse Transcription Mix II was used for complementary DNA synthesis. PCR reactions were conducted in a 10 µL volume, with the addition of 0.2 µL of complementary DNA to 2 × Color SYBR green qPCR Master Mix, all procured from EZBioscience. The QuantStudio 7 Flex real‐time PCR system was utilized for PCR, and a melting curve stage was included to assess primer specificity. Relative gene expression levels were calculated using the threshold cycle (2^−ΔΔ^ *^C^* ^t^) method. Relevant primers are listed as follows:

Gapdh: 5′‐ACCCAGAAGACTGTGGATGG‐3′ and 5′‐CACATTGGGGGTAGGAACAC‐3′; Batf3: 5′‐AGAAGGCTGACAAGCTCCACGA‐3′ and 5′‐CATCTTCTCGTGCTCCTTCAGC‐3′; CD300e: 5′‐CTGGGCAGAACCTGAGGATTAG‐3′ and 5′‐AGAGGCAGCTTCAGGAAGACCA‐3′; Adgre4: 5′‐GCTCTCCATCTGCCTTTTCCTG‐3′ and 5′‐GGAAGCCAAGTAGAGGTAGTGC‐3′; Lipg: 5′‐TACCTACACGCTGTCCTTTGGC‐3′ and 5′‐GCTCGCATTTCACCATCTCTGAG‐3′; Fabp4: 5′‐TGAAATCACCGCAGACGACAGG‐3′ and 5′‐GCTTGTCACCATCTCGTTTTCTC‐3′.

All these primers were synthesized by Tsingke Biotech Company (Beijing).

### 5.6 Immunofluorescence Staining

Mouse brains were dissected, fixed in 4% paraformaldehyde for 24 h at 4°C, and then sectioned into halves. After three washes in 0.01 M PBS, the halves were incubated with 15% sucrose for 24 h, followed by 30% sucrose for another 24 h for dehydration. The brains were frozen in OCT compound at −80°C and sectioned (16 µm) using a cryotome (Leica) at −20°C. Sections were treated with 0.1% Triton X‐100, blocked with 3% BSA in PBS for 1 h, and then incubated overnight at 4°C with primary antibodies (Iba1, Novus, NB100‐1028; CD68, abcam, ab125212; CXCR4, Affinity, AF5279; CXCL12, Proteintech, 17402‐1‐AP; Syndecan‐3, Proteintech, 10886‐1‐AP; PTN, Proteintech, 27117‐1‐AP; CD31, R&D, AF3628; F4/80, Abcam, ab90247) in 0.2% BSA. Secondary antibody (Invitrogen) staining was performed in 0.2% BSA at room temperature for 2 h. The sections were coverslipped using ProLong Diamond Antifade Mountant (Invitrogen) and imaged with an Olympus IXplore SpinSR microscope.

### 5.7 Preparation of Mouse Serum

Blood was collected from the ophthalmic vein of mice following anesthesia with isoflurane. The samples were then centrifuged at 2500 rpm for 15 min to separate the serum. The supernatant was carefully transferred into fresh tubes, flash‐frozen using liquid nitrogen, divided into aliquots, and preserved at −80°C for subsequent analysis.

### 5.8 Luminex Assay for Mouse Serum

The levels of 10 cytokines, including IL‐1β, TNF‐α, IFN‐γ, IL‐2, IL‐6, IL‐4, IL‐5, IL‐10, KC, and IL‐12p70, in mouse serum were detected by Luminex technology (Luminex‐X‐200, Bio‐Rad). The assay kit LX‐MultiDTM‐10 was provided by LabEX. Data were analyzed using MILLIPLEX Analyst software and quantified by standard curves. All procedures were performed according to the manufacturer's guidelines.

### 5.9 Flow Cytometry

Bone marrow cells were obtained by flushing the femurs and tibias of mice with PBS and filtering through a 70 µm nylon mesh to create a single‐cell suspension. After red blood cell lysis, the cells were blocked with anti‐mouse CD16/32 antibody (BioLegend, 101302) for 15 min, then stained with fluorescence‐conjugated antibodies for 30 min at 4°C in the dark. Antibodies were from BioLegend. The gating strategy is shown in Figure S3c. Samples were analyzed with a CytoFlex cytometer (Beckman Coulter) and FlowJo software version 10.4, collecting 50,000 events per sample.

### 5.10 Tissue Processing for Bulk RNA‐seq, RNA Extraction Library Construction, and Sequencing

Bone marrow cells were harvested from the femurs and tibias of mice, collected into tubes, flash‐frozen in liquid nitrogen, and stored at −80°C until RNA extraction. Total RNA was isolated using Trizol reagent (ThermoFisher), and its quality and concentration were evaluated using the Bioanalyzer 2100 system and the RNA 6000 Nano LabChip Kit (Agilent). Only RNA samples with an RNA Integrity Number (RIN) above 7.0 were selected for downstream processing. mRNA was purified from the total RNA, fragmented, and converted into cDNA through reverse transcription. Following second‐strand cDNA synthesis, A‐tailing, and adapter ligation were performed using dual‐index adapters. Size selection was carried out, and U‐labeled second‐strand cDNA was treated and amplified via PCR to generate cDNA libraries with an average insert size of 300 ± 50 bp. The libraries were then subjected to 2 × 150 bp paired‐end sequencing on an Illumina NovaSeq 6000 platform (LC‐Bio Technology) following the manufacturer's guidelines.

### 5.11 Processing of Bulk RNA‐seq Data

Paired‐end raw fastq files were merged into a single bam file using bedtools [71]. The processed BAM file was aligned to the reference genome (GRCm38) using STAR (version 2.7) with the default settings. featureCounts (version 1.6.0) was used to generate gene‐level read counts with the following parameters: “featureCounts‐T 40‐p‐t exon‐g.” The final differential gene expression count matrix was calculated using DESeq2 (version 1.28.1).

### 5.12 GO and GSEA Enrichment of Bulk RNA‐seq Data

GO analysis is performed using top DEGs (top 200 genes, *p*.adj < 0.05, log2FoldChange > 1). GO enrichment is calculated using the R package clusterProfiler. GSEA enrichment of all DEGs in each group is calculated using the R package fgsea.

### 5.13 Tissue Processing for scRNA‐seq

To minimize batch differences, bone marrow and brain samples from male 18‐month CD (LCA) (*n* = 3) and male 18‐month HFD (LHA) (*n* = 3) were isolated from the same mouse. scRNA‐Seq was performed by Novel‐Bio Bio‐Pharm Technology Co. Ltd. Bone marrow was flushed from the bones and stored in MACS Tissue Storage Solution (Miltenyi Biotec). After sieving and centrifugation, bone marrow cells were then filtered, centrifuged, and treated with a red blood cell lysis buffer (Miltenyi Biotec) to remove erythrocytes. Brain tissues were finely minced, enzymatically digested, filtered, and similarly processed to eliminate red blood cells. Cell viability was evaluated using the Countstar Fluorescence Cell Analyzer, and viable cells were isolated using the MACS Dead Cell Removal Kit (Miltenyi Biotec) to ensure high‐quality samples for sequencing.

### 5.14 scRNA‐seq Library Preparation and Sequencing

scRNA‐seq libraries were prepared using the Chromium Next GEM Single‐Cell 3’ GEM, Library & Gel Bead Kit v3 (PN‐1000094), following the manufacturer's instructions. Single‐cell suspensions derived from brain and bone marrow samples were loaded onto the Chromium single‐cell controller to create emulsions encapsulating individual cells and gel beads. Cells were lysed within the droplets, and the released RNA was barcoded through reverse transcription. The resulting cDNA was amplified, and sequencing libraries were subsequently constructed. Libraries were sequenced on the Illumina NovaSeq 6000 platform using 150 bp paired‐end reads. Raw sequencing data were processed using Cell Ranger (v.2.1.0) with default parameters aligned to the mouse reference genome mm39.

### 5.15 Data Preprocessing and Clustering

Seurat V3 (version 3.6.3) in R was used for downstream analysis [72]. The 10x Genomics digital gene expression matrix was normalized using the “NormalizeData” function with a scale factor of 10,000. Cells with fewer than 500 genes or a high proportion of mitochondrial genes (> 15%) were excluded. Principal component analysis was performed with the “RunPCA” function. Clustering was achieved using the “FindNeighbors” and “FindClusters” functions, considering the top 20 dimensions and a resolution of 1. Marker genes for each cluster were identified using the “FindAllMarkers” function with a minimum percentage of 0.25 and a log fold change threshold of 0.25. Dimensionality reduction was conducted using the “RunUMAP” function, and cell types were annotated based on canonical cell‐type‐specific marker genes.

### 5.16 DEGs Analysis and Gene Set Function Enrichment

Differential gene expression analysis of specific cell types was conducted using the FindMarkers function in Seurat, setting the log fold change threshold to 0 and the minimum percentage to 0. We filtered DEGs in each cell type using an absolute log fold change threshold > 0.5 and adjusted *p* < 0.05. Gene function enrichment analysis was performed using the clusterProfiler package with default settings [73]. GSVA was carried out based on DEGs identified in bone marrow and brain using the GSVA R package.

### 5.17 Ligand–Receptor Analysis

For ligand–receptor analysis, potential receptor–ligand pairs in specific cell types were enriched using CellChat (version 1.4.0) [38]. Receptors and ligands expressed in over 10% of cells within each cluster underwent further analysis. Communication patterns involving fewer than 10 cells in secreted signaling were excluded, with a cutoff determined by mean expression > 0.05 and *p* < 0.05. The strength of cell–cell interactions was indicated by the sum of receptor–ligand pairs in each cell–cell pairing. The Cxcl signaling network was visualized using both circle and dot plots.

### 5.18 Transcription Factors Enrichment Analysis

Transcription factor (TF) enrichment analysis was performed using SCENIC [45]. AUCell score matrices were combined with orthologous TFs, and TF modules were identified based on the connection specificity index (CSI). Heatmaps were used for visualization.

### 5.19 Statistical Analysis

Data were analyzed using GraphPad Prism (v8.2.1) software for statistical significance. The *p* value was determined by the Student's *t*‐test for two‐group comparisons. Each dot stands for a biological replication. The error bar represents the standard error of the mean.

## Author Contributions

Y.P., F.Y., and J.G. designed the research and interpreted the results. Y.P. and S.Z. conducted animal experiments and histological analysis. Y.P. conducted cell culture and RT‐qPCR. Y.P. and F.Y. performed and analyzed RNA‐seq. P.D. performed and analyzed flow cytometry. Y.P., F.Y., J.G., and J.Y. prepared the manuscript. S.Z. and Y.Z. provided essential support on animal experiments. F.Y., C.Z., J.G., and J.Y. supervised the study. All authors have read and approved the final manuscript.

## Ethics Statement

All experiments involving animals were conducted according to the ethical policies and procedures approved by the Animal Care and Use Committee of Shanghai Sixth People's Hospital (Approval no. DWLL2022‐0590).

## Conflicts of Interest

The authors declare no conflicts of interest.

## Supporting information




**Figure S1**: Transcriptomic and histological analysis of osteoclasts of LCA and LHA. **Figure S2**: Data quality. **Figure S3**: Verification of gene perturbation and serum cytokine level of LCA and LHA mice. **Figure S4**: Overlap perturbation genes between osteocyte‐deficient mice and LHA bone marrow samples. **Figure S5**: Single‐cell gene alteration and neural phenotype of brain by LHA.

## Data Availability

The scRNA‐Seq data have been deposited into the GEO repository with accession codes GSE217560. Bulk RNA‐Seq data have also been deposited into the GEO repository with accession codes GSE218105. Additional data supporting the findings of this study are available from the corresponding author upon request, and source data are provided with the paper.
