# Supplementary material for: Long‐Term High‐Fat Diet Affected Bone Marrow Microenvironment During Aging at Single‐Cell Resolution
Source: MedComm (2020). 2025 Jul 21;6(8):e70276. doi: 10.1002/mco2.70276 (PMC12277656; doi:10.1002/mco2.70276)
Supplement: Supplementary file 1 — Figure S1: Transcriptomic and histological analysis of osteoclasts of LCA and LHA. Figure S2: Data quality. Figure S3: Verification of gene perturbation and serum cytokine level of LCA and LHA mice. Figure S4: Overlap perturbation genes between osteocyte‐deficient mice and LHA bone marrow samples. Figure S5: Single‐cell gene alteration and neural phenotype of brain by LHA. [file MCO2-6-e70276-s001.pdf]

Supplementary Information

Title: Long-term high-fat diet affected bone marrow microenvironment during aging at single-cell resolution

Authors: Yidan Pang[1,2#], Siyuan Zhu[3#], Peng Ding[1,2#], Senyao Zhang[1,2], Yi Zhang[4], Fang Ye[5, 6*], Changqing Zhang[1*], Junjie Gao[1,2*], Jimin Yin [1*]

[1]Department of Orthopaedics, Shanghai Sixth People's Hospital Affiliated to Shanghai Jiao Tong University School of Medicine, Shanghai, 200233, China.
[2]Institute of Microsurgery on Extremities, and Department of Orthopedic Surgery, Shanghai Sixth People's Hospital Affiliated to Shanghai Jiao Tong University School of Medicine, Shanghai 200233, China.
[3]Department of General surgery, Shanghai Sixth People's Hospital Affiliated to Shanghai Jiao Tong University School of Medicine, Shanghai, 200233, China.
[4]Shanghai Diabetes Institute, Shanghai Key Laboratory of Diabetes Mellitus, Shanghai Clinical Centre for Diabetes, Shanghai Sixth People's Hospital Affiliated to Shanghai Jiao Tong University School of Medicine, Shanghai, 200233, China.
[5]Center for Stem Cell and Regenerative Medicine, Zhejiang University School of Medicine, Hangzhou 310058, China.
[6]Liangzhu Laboratory, Zhejiang University, 1369 West Wenyi Road, Hangzhou, China.

Corresponding Author
[#]These authors contributed equally.
[*]To whom correspondence should be addressed:
Fang Ye: 11618108@zju.edu.cn;
Changqing Zhang: zhangcq@sjtu.edu.cn;
Junjie Gao: colingjj@163.com;
Jimin Yin: dryinjimin@yeah.net

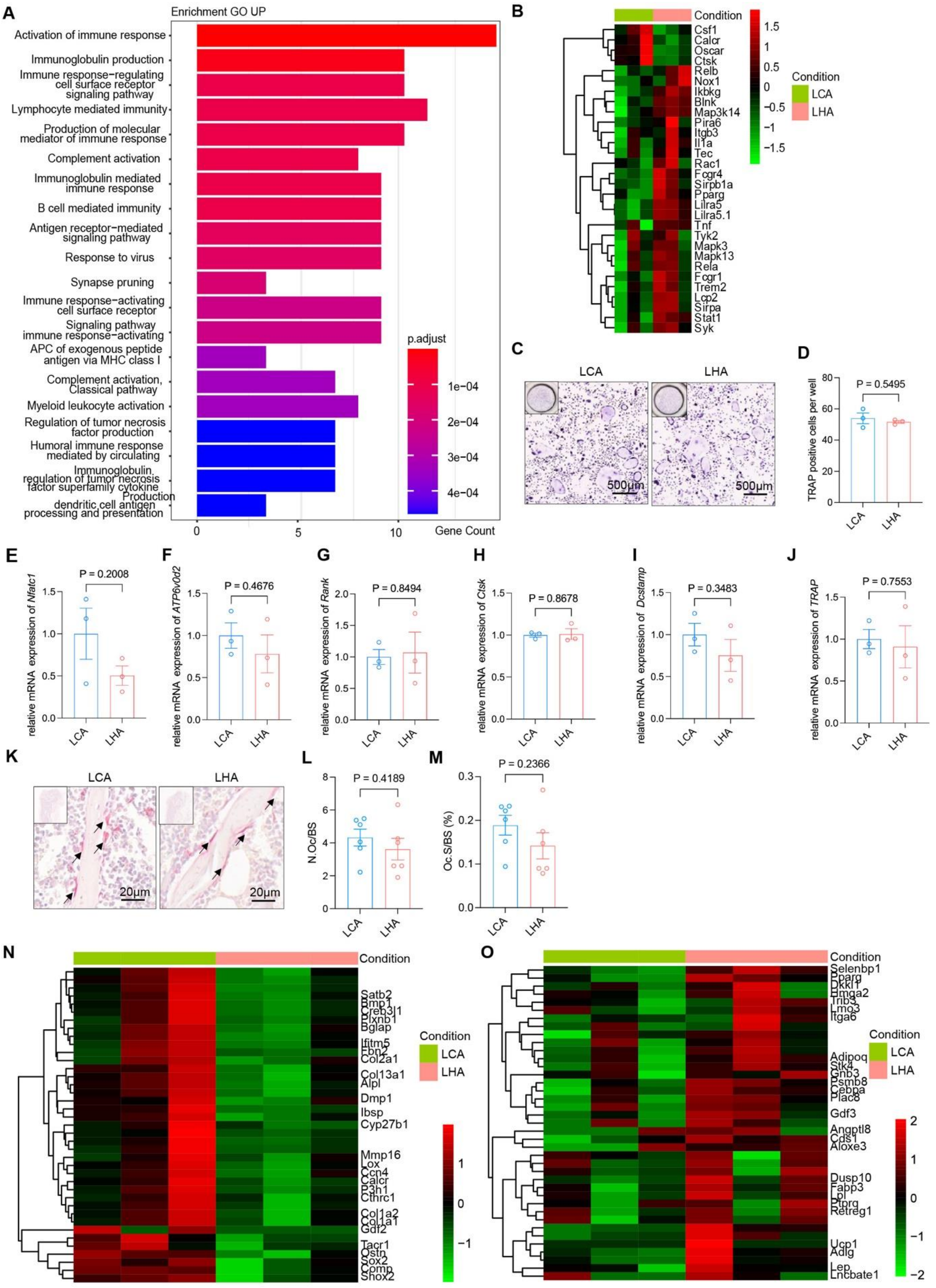

Figure S1 Transcriptomic and histological analysis of osteoclasts of LCA and LHA. A GO pathway analysis of up-regulated genes. **B** Gene expression heatmap of osteoclasts genes. **C** TRAP staining of osteoclastogenesis from CD and HFD mice and **D** quantification of TRAP positive cells per well. **E-J** RT-qPCR quantification of expression of the signature genes of osteoclasts. n=3. **K** TRAP staining of mice femur and **L-M** histomorphometry analysis of osteoclast surface and numbers per bone surface (N.Oc/BS and Oc.s/BS). n=6. **N** Gene expression heatmap showing significant down-regulation of ossification genes. **O** Gene expression heatmap showing enrichment of fat cell differentiation genes. Data are represented as mean ± SEM. Each dot represents a biological replicate.

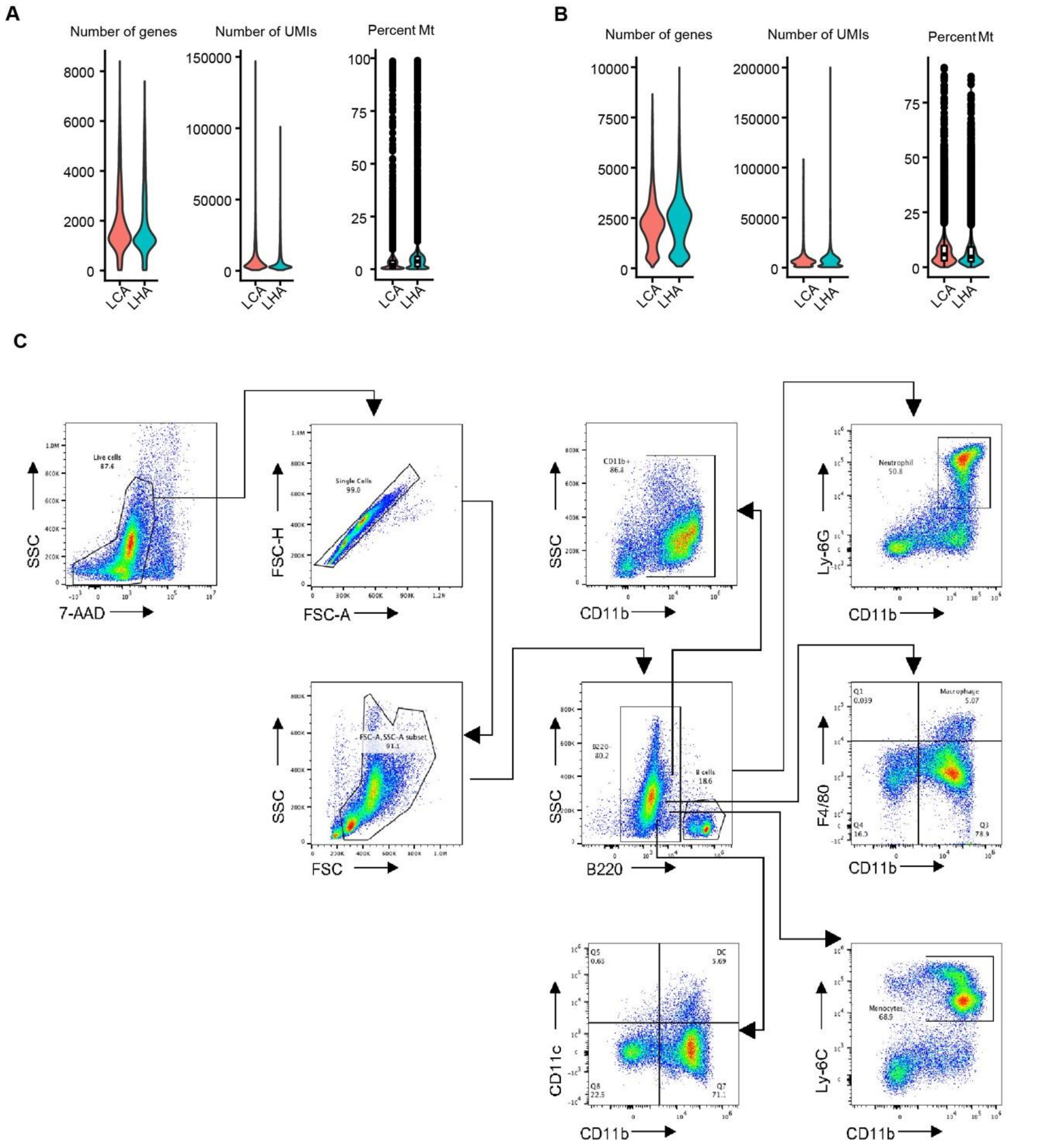

Figure S2 Data quality. A B Quality control of single-cell RNA-seq data in merged bone marrow **A** and brain **B** samples. **C** Gating strategy of bone marrow flow cytometry.

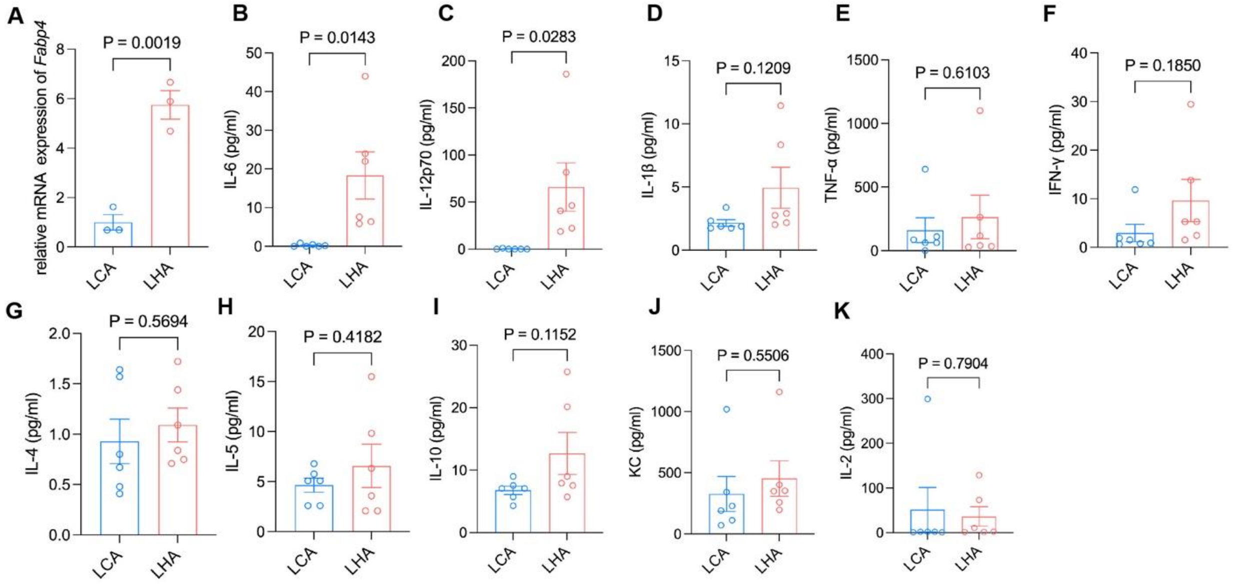

Figure S3 Verification of gene perturbation and serum cytokine level of LCA and LHA mice. A RT-qPCR of *Fabp4* expression level in bone marrow derived macrophages. **B-K** Luminex assay for mice serum levels of IL-1β, TNF-α, IFN-γ, IL-2, IL-6, IL-4, IL-5, IL-10, KC, IL-12p70. Data are represented as mean ± SEM. Each dot represents a biological replicate. n=6.

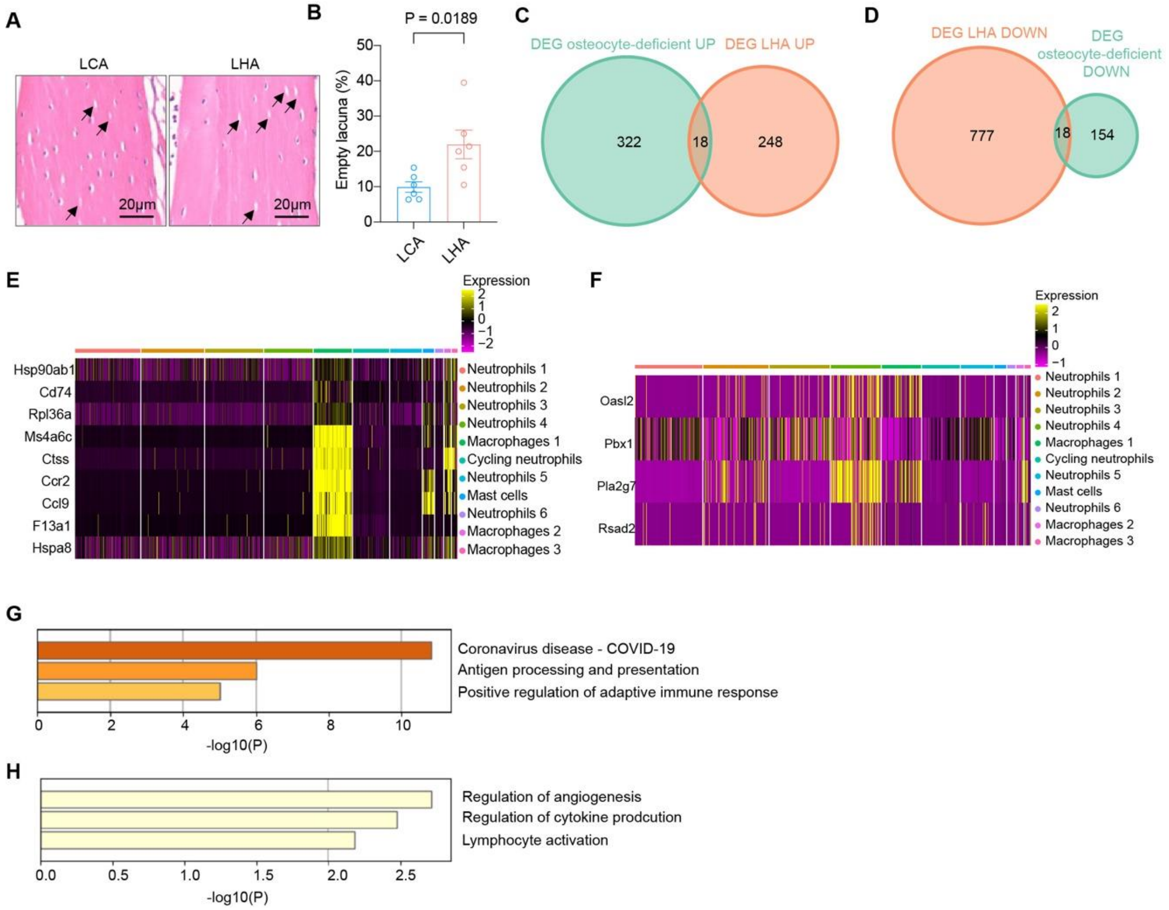

Figure S4 Overlap perturbation genes between osteocyte-deficient mice and LHA bone marrow samples. A HE staining of mice femur cortical bone and **B** quantification of empty lacuna. n=6. **C** Venn diagram of overlap up-regulated genes between osteocyte-deficient mice and HFD bone marrow samples. **D** Venn diagram of overlap down-regulated genes between osteocyte-deficient mice and HFD bone marrow samples. **E** Heatmap showing expression pattern of up-regulated overlapping genes in myeloid cells. **F** Heatmap showing expression pattern of down-regulated overlapping genes in myeloid cells. **G** Function enrichment of overlapped up-regulated genes. **H** Function enrichment of overlapped down-regulated genes.

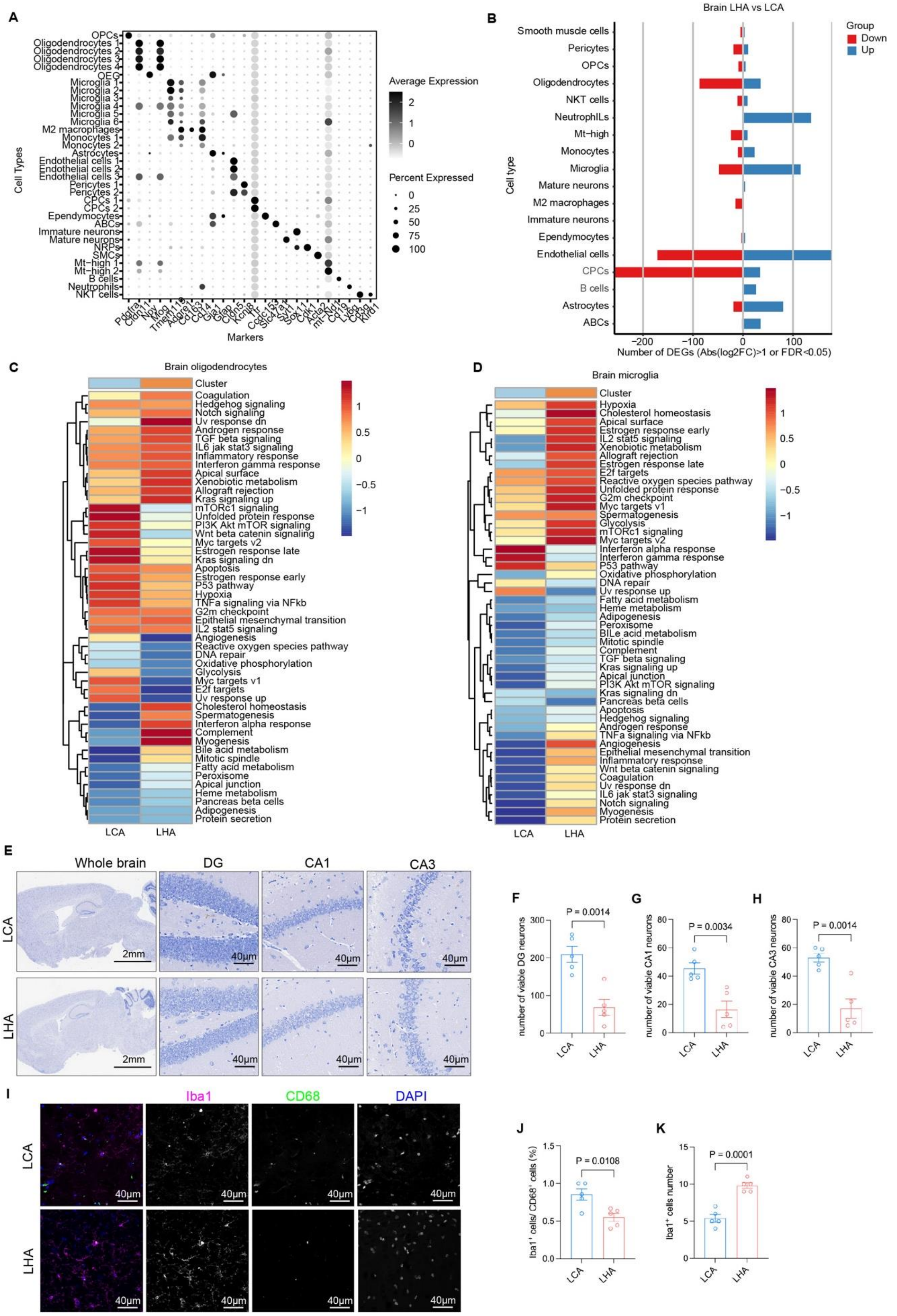

Figure S5 Single-cell gene alteration and neural phenotype of brain by LHA. A Dot plots showing the expression of marker genes in merged brain samples. **B** Bar plots showing the number of DEGs in brain in LHA condition. **C** GSVA enrichment results of brain oligodendrocytes and **D** brain microglia. **E** Nissl staining in the DG, CA3 and CA1 regions of the mouse hippocampus and **F-H** quantification of viable neurons in the DG, CA3 and CA1 regions. n=5. **I** Immunofluorescence staining of Iba⁺ and CD68⁺ cells in hippocampus and **J-K** quantification of number of Iba+ and CD68+ cells. n=5. Data are represented as mean ± SEM. Each dot represents a biological replicate.

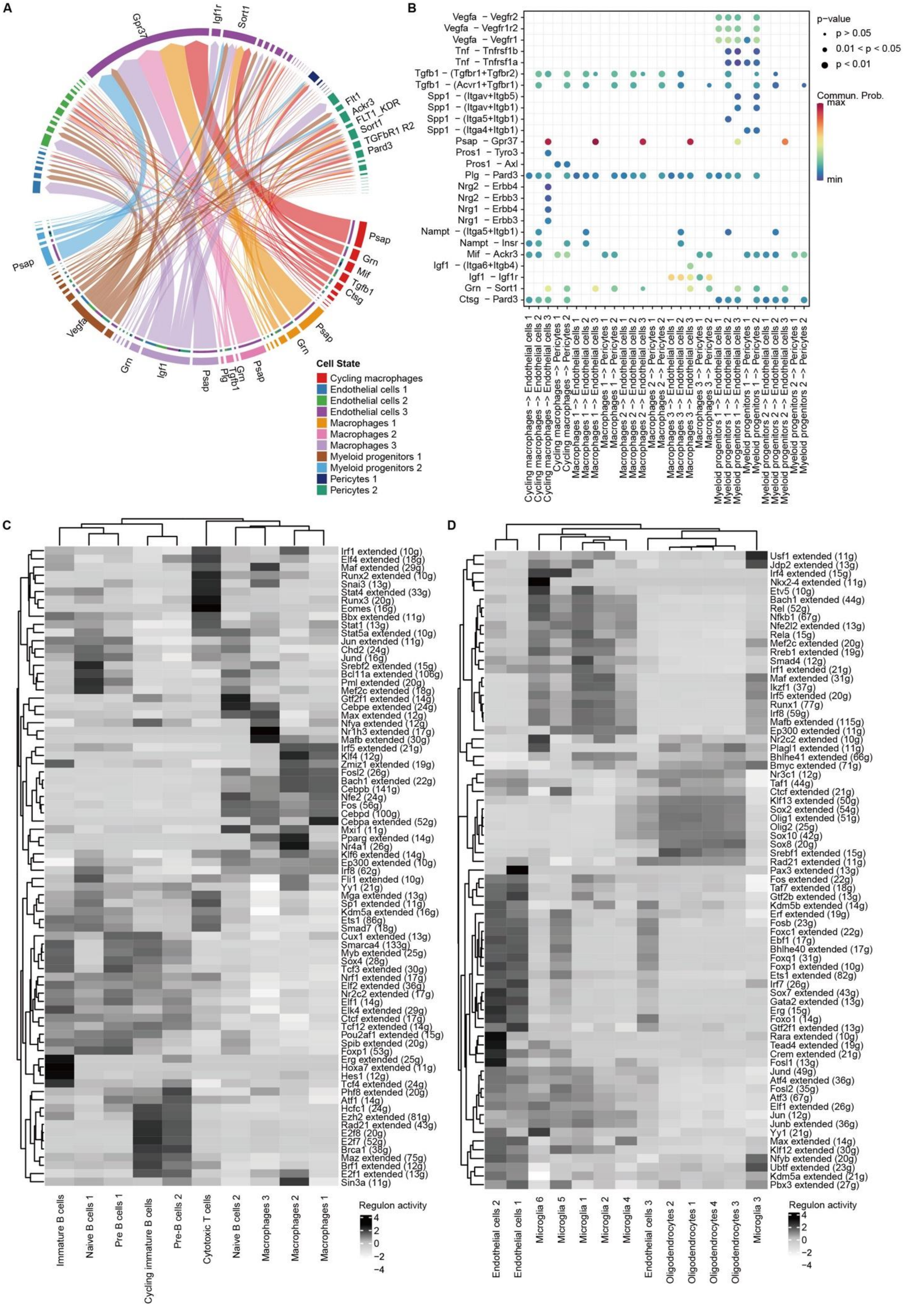

Figure S6 Ligand-receptor pairs between bone marrow and brain in LHA condition. A Cell-cell interaction network of overall ligand-receptor pairs between bone marrow myeloid cells and brain stromal cells (directions indicated the ligand-receptors from bone marrow to brain). **B** Heatmap showing the specific ligand-receptor pairs in **A**. **C**, **D** Heatmaps showing the critical TF regulon activity in bone marrow **C** and brain **D**.
